# Survival processing occupies the central bottleneck of cognitive processing: A psychological refractory period analysis

**DOI:** 10.3758/s13423-023-02340-z

**Published:** 2023-08-11

**Authors:** Meike Kroneisen, Edgar Erdfelder, Rika Maria Groß, Markus Janczyk

**Affiliations:** 1 Department of Psychology, Rheinland Pfälzische Technische Universität Kaiserslautern Landau, Landau, Germany; 2 Department of Psychology, University of Mannheim, Mannheim, Germany; 3 Department of Psychology, University of Bremen, Bremen, Germany

**Keywords:** Survival processing, Dual tasks, PRP, Bottleneck model, Effect propagation

## Abstract

Words judged for relevance in a survival situation are remembered better than words judged for relevance in a nonsurvival context. This survival processing effect has been explained by selective tuning of human memory during evolution to process and retain information specifically relevant for survival. According to the richness-of-encoding hypothesis the survival processing effect arises from a domain-general mechanism—namely, a particularly rich and distinct form of encoding. This form of information processing is effortful and requires limited cognitive capacities. In our experiment, we used the well-established psychological refractory period framework in conjunction with the effect propagation logic to assess the role of central cognitive resources for the survival processing effect. Our data demonstrate that the survival memory advantage indeed relies on the capacity-limited central stage of cognitive processing. Thus, rating words in the context of a survival scenario involves central processing resources to a greater amount than rating words in a nonsurvival control condition. We discuss implications for theories of the survival processing effect.

**Supplementary Information:**

The online version contains supplementary material available at 10.3758/s13423-023-02340-z.

According to the functionalist perspective, human memory has been shaped by evolutionary processes so that our ancestors were able to solve major adaptive problems during the Pleistocene (Nairne, 2005; Nairne et al., 2019; Nairne & Pandeirada, 2010). Therefore, memory systems should be sensitive to the content of to-be-stored information. To test this, Nairne et al. (2007) developed the survival processing procedure. Here, participants are instructed to either imagine being stranded on the grasslands of a foreign land and having to master several survival problems or to imagine themselves in a control scenario unrelated to survival, such as planning to move abroad and having to solve associated problems. Participants then rate a series of concrete words in terms of their relevance to the scenario. After a short distractor task, an unexpected free-recall test follows, typically resulting in a significant memory advantage for word material processed in the context of a survival scenario (e.g., Kazanas & Altarriba, 2015; Nairne et al., 2007). This phenomenon is referred to as the survival processing effect.

Following the pioneering work of Nairne and collaborators, the robustness and strength of the survival processing effect has been established in various ways. First, this effect has been replicated repeatedly, resulting in meta-analytic effect-size estimates ranging from η_p_^2^ = .06 to η_p_^2^ = .09 in between-subjects designs (Scofield et al., 2018).^1^ Second, although most studies used Nairne et al.’s (2007) relevance rating task to elicit survival processing during encoding, some authors also employed alternative survival processing instructions successfully—for example, generation of possible object uses (Kroneisen & Erdfelder, 2011, Exp. 3; Nairne et al., 2019) or choice of the most useful object for a scenario (Coverdale et al., 2019; Forester et al., 2020b). Third, replacing the moving control scenario by more exciting control scenarios does not eliminate the survival processing advantage (Bell et al., 2013; Kang et al., 2008; Kroneisen et al., 2022; Otgaar et al., 2011). Fourth, survival processing has been shown to outperform established memory-enhancing encoding techniques such as intentional learning, item generation from cues, self-reference ratings, or visual imagery (Kroneisen et al., 2013; Nairne et al., 2008).

Although the survival processing effect is robust, it may diminish or even vanish when instructions are manipulated in a way that weakens thoughts about object functions during the rating task. For instance, replacing the relevance rating task by an interactive imagery rating task (Kroneisen et al., 2013) or asking for ratings of threat instead of relevance (Bell et al., 2015) counteracts the benefit of survival processing. Supported by these and additional findings (e.g., Forester et al., 2019, 2020a, b), one prominent account to explain the effect assumes rich and distinct encoding induced by the encoding task (richness-of-encoding hypothesis; see Erdfelder & Kroneisen, 2014; Kroneisen & Erdfelder, 2011; Röer et al., 2013). Specifically, rating the relevance of objects in a grassland-survival scenario encourages participants to think about many unique object functions, both typical and atypical in nature, thereby stimulating highly distinctive ideas of how an item can be used to increase chances of survival. These ideas may later serve as powerful retrieval cues in the free recall test. By contrast, shifting encoding processes away from thoughts about possible object functions or even suppressing such thoughts reduces the survival processing effect (Bell et al., 2015; Kroneisen & Erdfelder, 2011; Kroneisen et al., 2013, 2021, 2022).

Most important for our present study, elaboration of possible object uses and relevance argument generation are effortful processes that require focused attention. Therefore, the survival processing advantage should diminish or even vanish when working memory resources are scarce. In line with this, individuals with less working memory resources, such as older adults, typically show a smaller or even no survival processing effect (Nouchi, 2012; Otgaar et al., 2015; Stillman et al., 2014). To test whether a task involves central cognitive resources, dual task experiments have often been used. If a primary task involves central cognitive resources, attention-demanding secondary tasks should interfere with task performance. Following this logic, Nouchi (2013), Kroneisen et al. (2014), and Stillman et al. (2014) examined the influence of a simultaneous secondary task on the survival processing effect. Results revealed that the cognitive resources devoted to the secondary task are crucial for the survival advantage. Specifically, if participants are required to continuously update and retain information in a parallel secondary task, the survival processing effect diminishes (Kroneisen et al., 2016).

A larger involvement of attentional resources is also suggested by longer rating response times (RTs) for survival relevance ratings than for control conditions (e.g., Kang et al., 2008; Kostic et al., 2012; Kroneisen et al., 2014, 2016; Kroneisen & Erdfelder, 2011). However, this result has not been uniformly observed (see, e.g., Burns et al, 2013; Nairne et al, 2007; Nouchi & Kawashima, 2012; Yang et al., 2014). One purpose of our current experiment is to assess the replicability of longer survival rating RTs and to gain some insights into possible moderators of this effect. Moreover, in those studies in which significantly longer survival rating RTs were observed, it remains unclear so far whether the RT difference between conditions is due to limited-capacity central processes or due to automatic processes (e.g., mind wandering stimulated by the scenario). In sum, while the dual task experiments of Kroneisen et al. (2014, 2016) and Nouchi (2013) along with correlational evidence reported by Nouchi (2012), Otgaar et al. (2015), and Stillman et al. (2014) suggest an important role of central cognitive resources in survival processing, the limited-capacity nature of underlying processes has never been tested directly.

Our current study extends prior research by asking (1) whether longer rating RTs in the survival condition can also be obtained in a Psychological Refractory Period (PRP) experiment, a more informative dual task setup common to the domain of multitasking research, and (2) whether this RT effect involves a general limited-capacity central resource which is also required in processes such as response selection. Previous evidence suggests that encoding items into and switching items in working memory indeed relies on general central resources (Janczyk, 2017; Jolicoeur & Dell’Acqua, 1998). Hence, a similar involvement of central resources might apply to survival processing as well.

PRP experiments (Pashler, 1994; Telford, 1931; see Fischer & Janczyk, 2022, for a recent review) are standard methods to assess effects of dual tasking. In modern versions, participants perform two tasks consecutively during each trial. These tasks involve separate stimuli (S1 and S2) that require two distinct responses (R1 and R2). In both tasks, RTs are measured from S1 or S2 onset until the respective response is made in Task 1 (RT1) and Task 2 (RT2). The critical manipulation in such experiments is the time between S1 and S2 onset, the stimulus onset asynchrony (SOA), which can either be short or long. With a short SOA, both tasks overlap in time, while this overlap becomes less the longer the SOA. Across many different stimuli, responses, and procedures, RT1 is often, though not always, unaffected by the SOA variation, while RT2 becomes much longer the shorter the SOA is, and thus the more both tasks overlap in time. This latter effect is called the PRP effect (Telford, 1931; for possible exceptions, see Janczyk et al., 2014).

The PRP effect is often explained by assuming a capacity limitation at a particular stage of processing. When one task already requires this capacity entirely, it is unavailable for other tasks. One of the most influential accounts in this regard is the central bottleneck model (Pashler, 1994; Welford, 1952). This model assumes that processing a task consists of three consecutive stages: The precentral (perceptual) stage, the central stage (often related to response selection; see also Janczyk & Kunde, 2020), and the postcentral (motor) stage. While the pre- and postcentral stages are assumed to run in parallel to all stages from other tasks, the critical assumption is that only one central stage can be processed at a time. This creates a bottleneck to which the model owes its name. What happens is that, if Task 2 finishes precentral processing while Task 1 is still occupying the central stage, central processing of Task 2 is postponed until the bottleneck is released again. This delay, called the *cognitive slack*, prolongs RT2. Importantly, this delay occurs with short SOAs, but less so (or not at all) with long SOAs (see Fig. 1a).

**Fig. 1:**
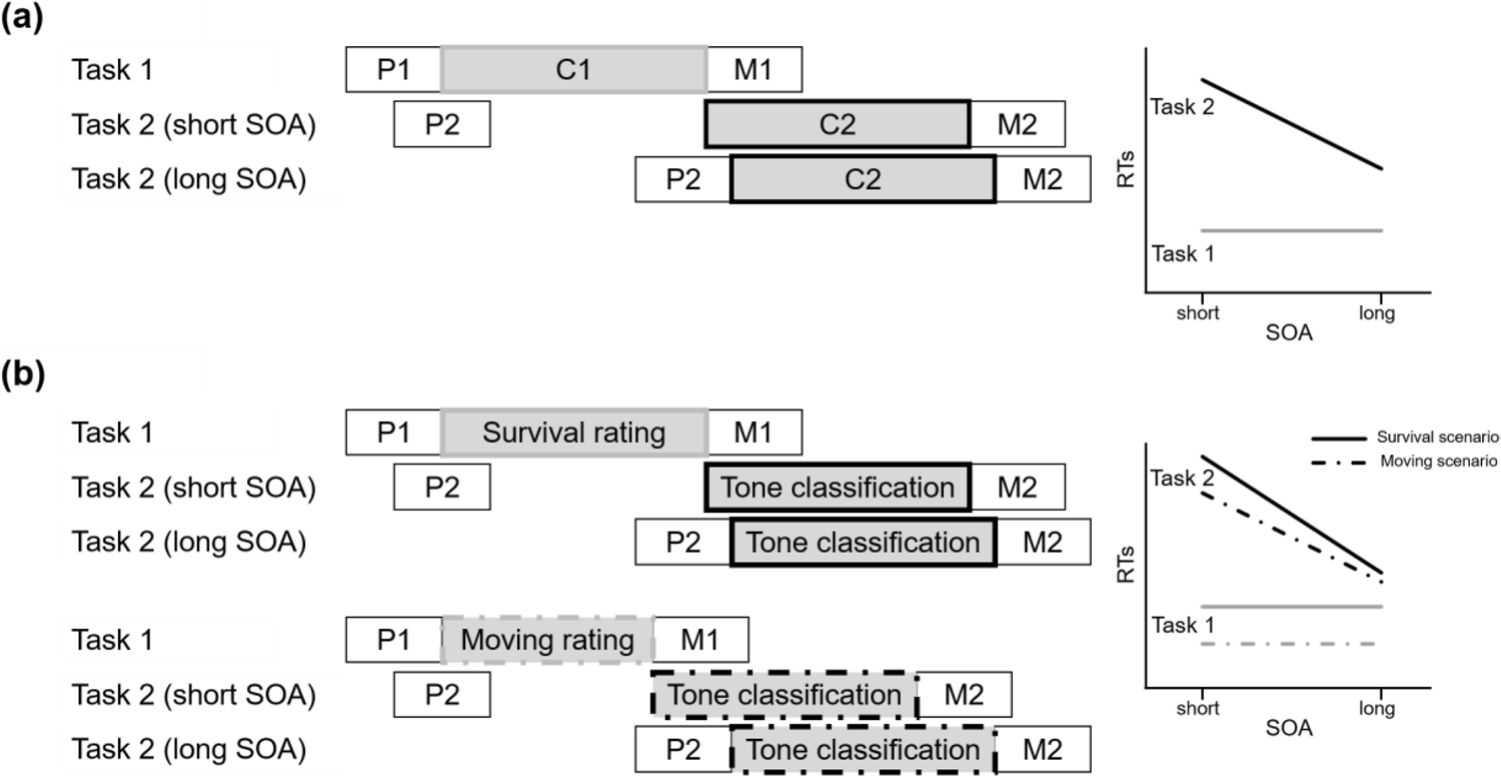
Illustration of the central bottleneck model and idealized predictions for RTs: **a** Processing of Task 1 and Task 2 for short and long SOA (stimulus onset asynchrony). **b** Processing of the survival versus the moving relevance rating (Task 1) and a tone classification task (Task 2) in the present experiment. For further explanations, please see the text. (P = precentral [perceptual] stage; C = central stage; M = postcentral [motor] stage)

To investigate whether a specific manipulation affects a processing stage (a) before or during the bottleneck or (b) after the bottleneck, the *effect propagation logic* can be used (see, e.g., Fischer & Janczyk, 2022; Janczyk et al., 2018, 2019; Kunde et al., 2012; Miller & Reynolds, 2003). Here, the manipulation of interest is implemented as Task 1 and an effect of this manipulation is expected in RT1. Importantly, for the short SOA condition, two predictions can be made for RT2, depending on the Task 1 stage responsible for the RT1 effect. First, if the manipulation of interest affects only the postcentral stage, it should not influence RT2, because this stage runs in parallel to the central stage of Task 2. Hence, Task 2 central processing does not need to be postponed. Second, if the manipulation affects the precentral or the central stage of Task 1 processing, the central stage of Task 2 must be postponed, and RT2s are affected in the same way as RT1s are. In other words, the effect in RT1 propagates into RT2.

This logic can also be exploited to investigate whether the survival scenario requires more central cognitive resources than control scenarios do. In this case, Task 1 would be the relevance rating and Task 2, for example, a simple tone classification task (see Fig. 1b). To test whether the survival scenario indeed requires more central resources, the most important comparison refers to the short SOA condition. If our hypothesis were true, the longer processing required for ratings in the survival compared with the moving condition leads to a corresponding postponement of the Task 2 central stage. Hence, the same RT1 difference between both scenarios should also be observed in RT2. Moreover, for sufficiently long SOAs, the postponement of Task 2 diminishes and hence the RT2 effect should become smaller or even vanish. Thus, theoretically, an interaction of SOA and the Task 1 manipulation is expected. However, such an interaction effect does not always manifest in a statistically significant way (see, e.g., Paelecke & Kunde, 2007, Exp. 3; Wirth et al., 2015, Exps. 2 and 4), because the size of this effect depends on the particular tasks, their RTs, and also the specific SOA levels used.

## Method

### Participants

The original sample consisted of 133 University of Mannheim students (mean age = 21.41 years, 97 female)—the maximum sample size obtainable within the predetermined data collection period. Participants either received course credit or a flat-rate monetary reimbursement as an incentive. One participant had to be excluded due to an experimental error, and another one was excluded outright for not having responded in Task 2 throughout the PRP experiment, leaving 65 and 66 participants in the moving and survival scenario, respectively. Further exclusions are reported below. All participants reported normal or corrected-to-normal vision and hearing and were naïve with regard to the underlying hypotheses.

### Apparatus, stimuli, and material

Stimuli in the relevance rating task were 60 concrete nouns selected from Experiment 1, 2, and 3 of Nairne et al. (2007) and translated to German. To absorb primacy and recency effects typically found in free recall, we added 12 buffer words, six at the beginning and six at the end of the list. Apart from the buffer words, all words were presented in random order. Stimuli in the tone classification task were 300- and 900-Hz tones presented for 50 ms. Tone classification responses were given by pressing the *x* and *y* key of a standard German keyboard. The tone–key mapping was determined randomly for each participant. Relevance ratings were provided by pressing the corresponding number key on the keyboard. Experiments were controlled by PCs running E-Prime 2.0 (Psychology Software Tools, Pittsburgh, PA, USA).

We used German translations of the standard survival and moving scenarios (as introduced by Nairne et al., 2007):

- Survival: *In this task, we would like you to imagine that you are stranded in the grasslands of a foreign land, without any basic survival materials. Over the next few months, you’ll need to find steady supplies of food and water and protect yourself from predators. We are going to show you a list of words, and we would like you to rate how relevant each of these words would be for you in this survival situation. Some of the words may be relevant and others may not—it’s up to you to decide.*
- Moving: *In this task, we would like you to imagine that you are planning to move to a new home in a foreign land. Over the next few months, you’ll need to locate and purchase a new home and transport your belongings. We are going to show you a list of words, and we would like you to rate how relevant each of these words would be for you in accomplishing this task. Some of the words may be relevant and others may not—it’s up to you to decide.*

### Design

We employed a 2 × 2 mixed design, with scenario as a between-subject factor (survival vs. moving) and stimulus onset asynchrony (SOA) between the stimuli of the rating and the tone classification task as a within-subject factor (100 ms vs. 1,000 ms). Free recall rates, relevance ratings, as well as RTs in the rating task (RT1) and the tone classification task (RT2) and the corresponding error rates in the tone classification task served as dependent variables. Note that the PRP approach mainly makes predictions concerning RTs. However, it is standard to analyze error rates in the respective tasks in addition (here: in the tone classification) to control for the possibility that a speed-accuracy tradeoff is responsible for an observed RT effect (Wickelgren, 1977).^2^

### Procedure

Participants were randomly assigned to the survival or the moving scenario. The whole experiment comprised three parts.

In Part 1, the PRP experiment took place, which included relevance ratings of the words. More precisely, each participant was instructed to read the respective scenario and then rate words with respect to their relevance for this scenario (Task 1: relevance rating task). Immediately following this instruction, participants received two practice trials for the rating task only. Next, each of the two tones was presented once, along with the instruction which key to press to each pitch (Task 2: tone classification task). This was followed by another two practice trials of the PRP experiment proper—that is, with both tasks and the respective stimuli separated by an SOA. Participants were explicitly instructed to respond as fast as possible in either task and to provide the relevance rating always prior to the tone classification response.

Each of the following 72 experimental trials (comprising 60 target words and the 12 primacy and recency buffer words) began with a centered fixation cross presented for 1,000 ms. The to-be-rated words were then presented individually in the center of the screen with a Likert-type relevance rating scale ranging from 1 (*not relevant*) to 5 (*very relevant*) underneath. Participants were asked to provide relevance ratings by pressing the corresponding number key (Task 1). Irrespective of how fast this rating was given, each word remained on the screen for exactly 5,000 ms to ensure comparable exposure times. For the tone classification task (Task 2), one of the two tones was presented following an SOA of 100 ms or 1,000 ms after word onset. Low- and high-pitch tones were equally frequent in both SOA conditions. The participants’ task was to classify tones as high- or low-pitched by pressing the respective response key. The next trial started again with a 1,000 ms fixation cross.

In case participants (a) failed to generate a relevance rating within 5,000 ms, (b) incorrectly responded in the tone classification task, or (c) provided the tone response prior to the relevance rating, they were cautioned to respond faster and/or in line with the instructions, respectively (an error message was shown for 1,000 ms).

In the subsequent Part 2 of the experiment, participants completed a short version of the complex span task (Rummel et al., 2019) as a distractor task for approximately 10 min. This was immediately followed by Part 3, in which memory test instructions appeared unexpectedly. Participants were asked to type all previously rated words (from the first part of the experiment) they could recall, regardless of the order of their presentation (i.e., free recall). A maximum of 8 min was allowed to complete this task.

The whole experiment took approximately 35 min to complete, after which participants were debriefed.

### Data preprocessing and statistical analyses

For all analyses, we first excluded invalid trials, that is, trials where participants corrected their rating or responded in the wrong order (9.8% of all trials; including these trials in the free recall analyses did not affect the results qualitatively). We further excluded eight participants with more than 15% errors in Task 2, leaving *N* = 123 for data analyses, 63 and 60 participants in the moving and the survival scenario, respectively. For RT analyses, trials with erroneous tone classifications were excluded as were additionally those in which RT deviated more than 2.0 standard deviations from the participants’ mean RT (calculated separately for the two SOA conditions) in either the rating or the tone classification task (5.04% of the trials).^3^

Mean (correct) RTs were submitted to a 2 × 2 mixed ANOVA, with SOA (100 vs. 1000 ms) as a within-subject factor and scenario (moving vs. survival) as a between-subject factor. For the theoretically most important analyses (see the Introduction), that is, to assess whether an effect of scenario was of the same size (or smaller) for Task 2 than for Task 1, data from the SOA = 100 ms condition were submitted to a 2 × 2 mixed ANOVA, with task (1 vs. 2) as a within-subject factor and scenario (moving vs. survival) as a between-subject factor. In addition, recall rates and ratings were compared between scenario groups using two-samples *t* tests. Finally, to assess the joint effects of scenarios and ratings on memory performance, a 2 × 5 mixed ANOVA on the free recall rates with scenario as a between-subject factor and rating category as a within-subject factor was conducted. Eight participants produced empty cells in this case and were excluded from this analysis accordingly.

### Statistical power

Sensitivity analyses (Erdfelder, 1984) with G*Power 3.1 revealed that, given α = .05 and a minimum power level of 1 − β = .80, our sample size *N* = 123 suffices to detect effects of medium size (more precisely, Cohen’s *d* = 0.51) or larger with two-tailed two-samples *t* tests (Faul et al., 2009). As outlined in the Introduction (see Footnote 1), this matches Scofield et al.’s (2018) meta-analytic estimate of the survival processing effect almost perfectly. Concerning the 2 × 2 mixed ANOVAs, sensitivity analyses with the same input specifications and an assumed correlation of ρ = .5 between SOA conditions showed that not only medium scenario effects (detectable Cohen’s *f* = .22), but also small SOA main effects and small scenario × SOA interactions can be detected with a total *N* = 112 (both detectable *f*s = .13). Notably, the 2 × 5 mixed ANOVA is sensitive to even slightly smaller *f*s under H_1_ (detectable *f* = .20 and *f* = .10 for between and within-subject effects, respectively). Hence, our experiment is sufficiently powered to test all hypotheses of interest.

## Results

### Recall performance

The mean proportion of correctly recalled words (from valid trials only, see above) was higher for the survival (*M* = 0.40, *SD* = 0.11) than for the moving scenario (*M* = 0.29, *SD* = 0.10), *t*(121) = 5.48, *p* < .001, *d* = 0.99.^4^

### Rating results

Mean relevance ratings were higher for the survival (*M* = 3.07, *SD* = 0.40) than for the moving scenario (*M* = 2.45, *SD* = 0.49), *t*(121) = 7.61, *p* < .001, *d* = 1.37.

### Recall as a function of scenario and relevance ratings

**Fig. 2:**
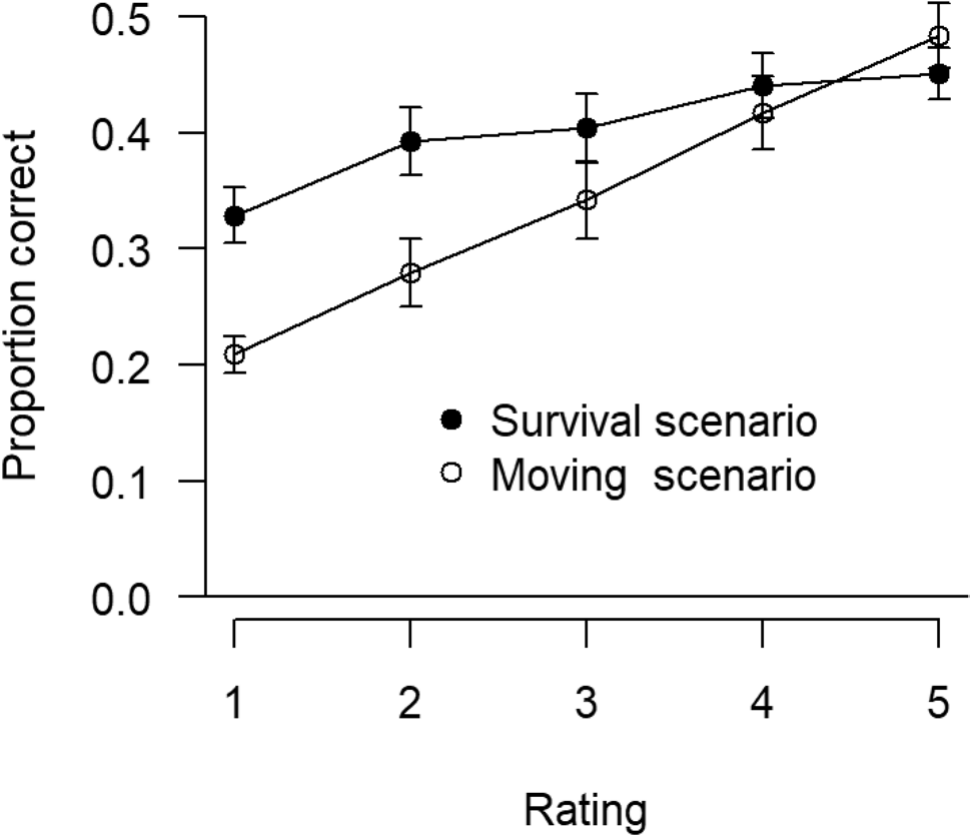
Mean proportions of correct recall for each scenario, separately for rating categories. Error bars indicate standard errors of the mean

Figure 2 illustrates survival and moving recall performance separately for relevance rating levels. The 2 × 5 mixed ANOVA revealed significant main effects of scenario, *F*(1, 113) = 7.39, *p* = .008, η_p_^2^ = .06, and rating level, *F*(4, 452) = 18.22, *p* < .001, η_p_^2^ = .14, Greenhouse–Geisser ε = .90, as well as a significant interaction, *F*(4, 452) = 3.04, *p* = .021, η_p_^2^ = .03, Greenhouse–Geisser ε = .90. Overall, free recall performance increases monotonically with relevance ratings (a congruity effect; see Butler et al., 2009), and the survival processing advantage is found for most rating levels but diminishes from low to high relevance ratings. This pattern replicates similar results we found in previous research (cf. Kroneisen & Erdfelder, 2011; Kroneisen et al., 2013, 2014, 2016, 2021, 2022).

### PRP analysis: Relevance rating task (Task 1)

**Fig. 3:**
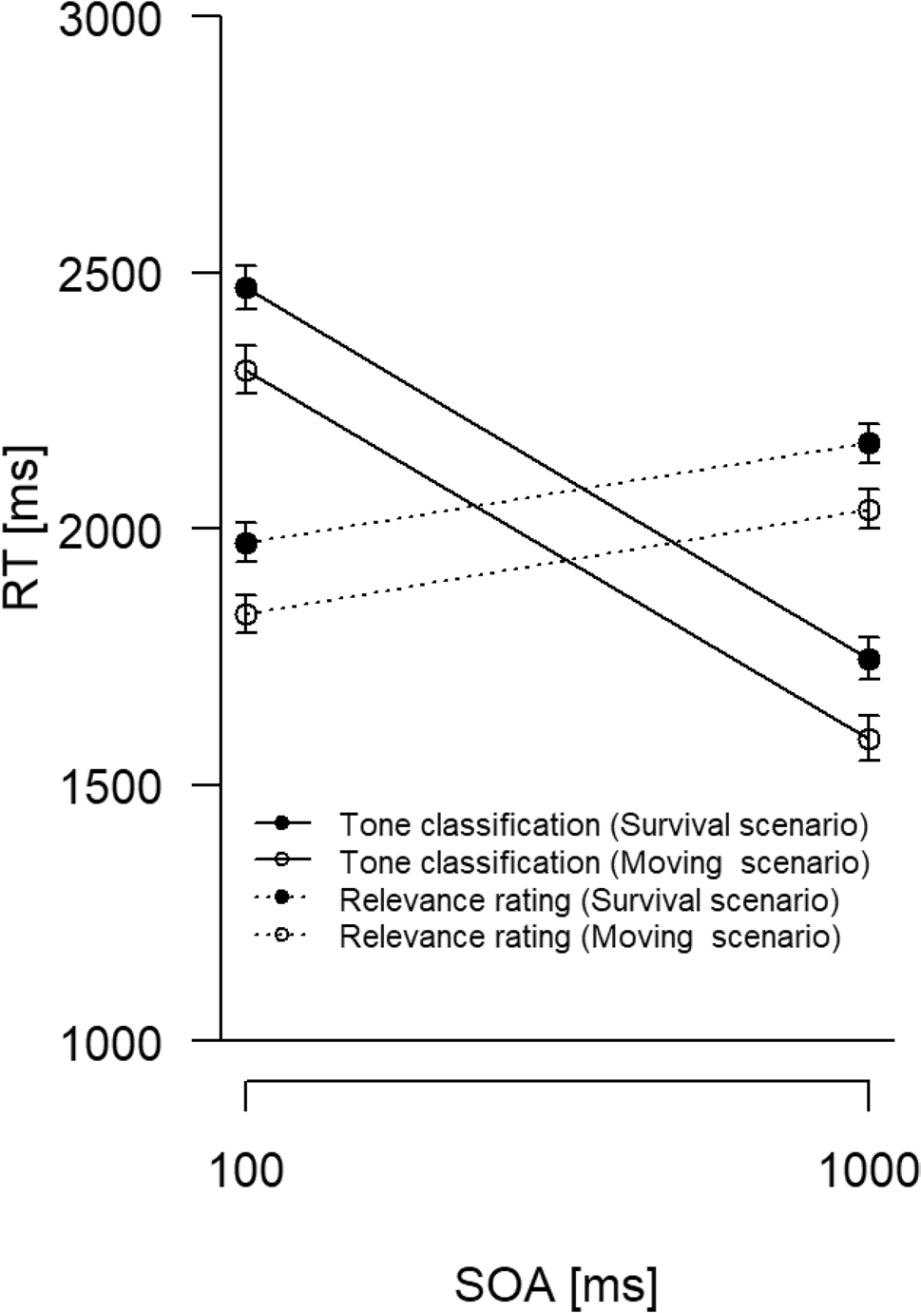
Mean (correct) RTs in milliseconds (ms) for the relevance rating task (Task 1) and the tone classification task (Task 2) as a function of stimulus onset asynchrony (SOA in ms) and scenario. Errors bars are standard errors of the means

Mean RTs for the relevance rating task as a function of SOA and scenario are visualized in Fig. 3 (dotted lines). RTs were longer in the survival compared with the moving scenario, *F*(1,121) = 7.23, *p* = .008, η_p_^2^ = .06, and with a long compared with a short SOA, *F*(1, 121) = 106.55, *p* < .001, η_p_^2^ = .47. The interaction was not significant, *F*(1, 121) = 0.08, *p* = .782, η_p_^2^ < .01.

### PRP analysis: Tone classification task (Task 2)

Mean correct RTs for the tone classification task as a function of SOA and scenario are visualized in Fig. 3 (solid lines). RTs were longer in the survival compared with the moving scenario, *F*(1, 121) = 7.37, *p* = .008, η_p_^2^ = .06. RTs were also longer with the short compared with the long SOA, thus a PRP effect, *F*(1, 121) = 1339.08, *p* < .001, η_p_^2^ = .92. The interaction was not significant, *F*(1, 121) = 0.02, *p* = .895, η_p_^2^ < .01. Mean error percentages were 3.62 and 2.24 for the moving scenario and 4.21 and 2.35 for the survival scenario (for the 100 vs. 1,000 ms SOA, respectively). Only the main effect of SOA on errors was significant, *F*(1, 121) = 12.53, *p* = .001, η_p_^2^ = .09; scenario: *F*(1, 121) = 0.39, *p* = .532, η_p_^2^ < .01; interaction: *F*(1, 121) = 0.29, *p* = .594, η_p_^2^ < .01.

### Comparison between tasks at the short SOA

Considering only the short SOA, the RT difference between both scenarios was descriptively larger for the tone classification task (161 ms) than for the relevance rating task (140 ms). As would be expected from the previous analyses, the corresponding ANOVA yielded significant main effects of task, *F*(1,121) = 790.80, *p* < .001, η_p_^2^ = .87, and of scenario, *F*(1, 121) = 7.38, *p* = .008, η_p_^2^ = .06. Of particular importance in the present context, the interaction was not significant, *F*(1, 121) = 0.37, *p* = .542, η_p_^2^ < .01. In other words, the effect of scenario on Task 1 (relevance rating) fully propagated into Task 2 RTs (tone classification).

## Discussion

The present experiment aimed at (a) replicating the survival processing effect and (b) testing whether the survival processing advantage goes along with a greater need of limited-capacity central resources. Previous dual task experiments already indicated that this might be the case (Kroneisen et al., 2014, 2016; Nouchi, 2013). Here, we used a PRP experiment and utilized the effect propagation logic (e.g., Miller & Reynolds, 2003) to further assess this. If survival processing indeed requires limited-capacity central resources, the RT effect obtained in the respective rating task should—at a short SOA—propagate into an unrelated Task 2, in our case a tone classification task, and be of the same size. An additional expectation is that, with a sufficiently long SOA, the postponement of Task 2 diminishes so that the effect on RT2 becomes smaller, resulting in an overadditive interaction of SOA and the Task 1 manipulation.

As expected, our results replicate the survival processing advantage relative to a moving scenario. Thus, the survival processing effect again proved to be a robust phenomenon. Also consistent with previous reports, recall performance increased monotonically with ratings (congruity effect; see Butler et al., 2009). Although relevance ratings were significantly higher in the context of the survival scenario compared to the moving scenario, congruity effects cannot explain the survival processing advantage, because the survival processing effect is still evident when relevance ratings are included as an additional factor into the analysis (see Fig. 2). In general, the survival advantage is largest for items receiving the lowest relevance ratings; it tends to decrease with increasing ratings and to vanish for the highest rating, but never becomes significantly negative, again replicating previous results (e.g., Kroneisen et al., 2013, 2014, 2016, 2021, 2022).^5^

Regarding the key hypothesis of this study, the significant difference of relevance rating RT1s (Task 1) between the survival and the moving scenario was obtained in the tone classification task RT2s (Task 2) as well, and it was of the same size at the short SOA. This is our most important result, because it shows that Task 2 is postponed until the central bottleneck (occupied by Task 1) is released again. We did not obtain a significant overadditive interaction between SOA and the Task 1 manipulation, though. While such an interaction is theoretically expected with a sufficiently long SOA, empirically it is not always obtained (e.g., Paelecke & Kunde, 2007; Wirth et al., 2015). In our case, a “long” SOA of 1,000 ms was probably still too short to yield a sufficiently strong interaction effect. However, together with the overall PRP effect obtained in Task 2, the observed propagation from Task 1 into Task 2 suggests that both tasks draw on the same limited cognitive resources and thus cannot be executed in parallel. Notably, this conclusion goes beyond the dual task experiments of Kroneisen et al. (2014, 2016) and Nouchi (2013) by showing that survival processing not only requires central cognitive resources, but also occupies them exclusively so that all other tasks requiring them must wait until survival processing has finished. In our experiment, the larger involvement of limited cognitive resources in survival processing translates into a significant increase of central processing time of about 150 ms relative to the moving control scenario.

In sum, the current study provides evidence that central cognitive resources are indeed used to a larger extent in survival processing than in control conditions. As outlined in the Introduction, this is in line with the richness-of-encoding hypothesis, because rich, elaborative forms of encoding, such as thinking about various object functions (cf. Bell et al., 2015), necessarily involve limited cognitive resources.

Also evident by our results, the memory advantage gained by survival processing is most pronounced for items judged to be of low relevance. An explanation consistent with the richness-of-encoding account is that rich forms of encoding are generally quite easy for items of high functional relevance, and this holds irrespective of the scenario that serves as the relevance criterion. For items judged less relevant, however, the survival scenario stimulates more elaborate encoding than the moving control scenario does, simply because participants engage more strongly and persistently in thinking about diverse functions of objects in a complex live-threatening context. If this explanation is correct, one would expect relevance rating RTs to be relatively short for high rating categories in general because detection of relevant object uses is easy for either scenario. With lower relevance judgments, however, differences in rating RTs between survival and control scenarios should increase, because participants engage more strongly and persistently to identify potentially useful object functions in the former context. As revealed by a reanalysis of our rating RT1s in the Online Supplemental Material, this is indeed what we observed.^6^ This supports our explanation why rating outcomes moderate the survival processing effect. It also suggests a mechanism that can potentially explain why RT differences between scenarios sometimes disappear: If rating distributions are shifted towards the higher ratings, RT differences between scenarios tend to diminish.

Although our results are generally in line with the richness-of-encoding account, one might argue that they conflict with other recently reported results. For instance, using a generation instead of a relevance rating procedure, Nairne et al. (2019) found that the survival processing advantage persists even if participants are asked to generate a single function of an object in the respective scenario only, thus, presumably inhibiting richness-of-encoding (see also Coverdale et al., 2019). We would maintain that the richness-of-encoding account does not predict the survival processing advantage to vanish under specific conditions, because it is generally difficult if not impossible to equate survival and control conditions in terms of richness of encoding. Rather, it predicts that the size of the survival processing effect is *moderated* by the number of possible object functions considered (note that this is an interaction hypothesis, not a simple main effect hypothesis). For example, the survival versus moving advantage should be larger when generating four object functions than when generating a single function, a result that we previously found (Kroneisen & Erdfelder, 2011, Exp. 3).

What are the implications of our current results for the functionalist perspective on human memory? Clearly, our results are at odds with narrow conceptions of evolutionary theories that subscribe to criteria such as encapsulation and automaticity of functionally specialized evolved modules (Fodor, 1983). According to our results, the mechanism driving the survival processing advantage is cognitively controlled, effortful, and attention-demanding rather than spontaneous and automatic. Moreover, as detailed above, the survival-processing advantage relies on a domain-general memory process—richness of encoding—that is not limited to a specific content domain. Notably, more recent functionalist perspectives on human cognition are compatible with such ideas (e.g., Nairne & Pandeirada, 2016; Pietraszewski & Wertz, 2022) so that there is no obvious conflict. The question rather is in which respect survival processing is special—a key research question in the functionalist research agenda. For instance, we have shown that cognitive activities that require central resources are postponed to a later point in time as long as survival processing is active. But what happens to survival processing when this task is initiated while another central processing task is still active? Is survival processing also postponed? Or is it prioritized such that it interferes with or even interrupts the active task instantly? Given our current results, we simply cannot tell. However, the PRP approach provides an appropriate framework for investigating this interesting follow-up research question in the future.

### Supplementary Information

Below is the link to the electronic supplementary material.

Supplementary file1 (DOCX 42 KB)

## Data Availability

The data and materials of the reported experiment are openly available via the Open Science Framework (OSF; https://osf.io/jk4xr/).

## Footnotes

1 This translates to Cohen’s *d* between 0.50 and 0.62 (i.e., at least a medium effect; cf. Cohen, 1988). As already shown by Cohen (1988), for two groups of equal size, *d* = 2 ⋅ $\sqrt{(\mathrm{\eta p2}/(1- \mathrm{\eta p2}))}$.

2 Previous results based on secondary tasks similar to Task 2 in the current experiment (cf. Kroneisen et al., 2014, 2016) suggest no difference in Task 2 error rates between both scenarios.

3 All data are available via the OSF and can be accessed online (https://osf.io/jk4xr/).

4 When the mean proportion of correctly recalled words was calculated relative to all 60 items each participant was presented with (instead of only the number of valid trials), the results remained virtually the same (survival scenario: *M* = 0.36, *SD* = 0.12; moving scenario: *M* = 0.27, *SD* = 0.10), *t*(121) = 4.53, *p* < .001, *d* = 0.82.

5 Of course, since relevance ratings are selected by participants and therefore observational in nature, their effects cannot be interpreted as easily as those of randomized factors. For example, frequencies of rating categories may differ between participants, and item attributes (e.g., word frequency, familiarity) may vary between rating levels. Thus, as in case of other observational factors, confounds cannot be ruled out on a priori grounds. However, this does not invalidate the well-supported empirical claim that the survival processing advantage tends to increase with decreasing relevance ratings.

6 We thank an anonymous reviewer for suggesting this informative analysis.

## References

[CR1] Bell R, Röer JP, Buchner A (2013). Adaptive memory: The survival processing advantage is not due to negativity or mortality salience. Memory & Cognition.

[CR2] Bell R, Röer JP, Buchner A (2015). Adaptive memory: Thinking about function. Journal of Experimental Psychology. Learning, Memory, and Cognition.

[CR3] Burns DJ, Hart J, Griffith SE, Burns AD (2013). Adaptive memory: The survival scenario enhances item-specific processing relative to a moving scenario. Memory.

[CR4] Butler AC, Kang SHK, Roediger HL (2009). Congruity effects between materials and processing tasks in the survival processing paradigm. Journal of Experimental Psychology: Learning, Memory, and Cognition.

[CR5] Cohen J (1988). Statistical power analysis for the behavioral sciences.

[CR6] Coverdale ME, Pandeirada JNS, Nairne JS (2019). Survival processing in a novel choice procedure. American Journal of Psychology.

[CR7] Erdfelder E (1984). Zur Bedeutung und Kontrolle des beta-Fehlers bei der inferenzstatistischen Prüfung log-linearer Modelle [On significance and control of the beta error in statistical tests of log-linear models]. Zeitschrift für Sozialpsychologie.

[CR8] Erdfelder E, Kroneisen M, Schwartz BL, Howe M, Toglia M, Otgaar H (2014). Proximate cognitive mechanisms underlying the survival processing effect. What is adaptive about adaptive memory?.

[CR9] Faul F, Erdfelder E, Buchner A, Lang AG (2009). Statistical power analyses using G*Power 3.1: Tests for correlation and regression analyses. Behavior Research Methods.

[CR10] Fischer R, Janczyk M, Kiesel A, Johannsen L, Koch I, Müller H (2022). Dual-task performance with simple tasks. Handbook of human multitasking.

[CR11] Fodor J (1983). The modularity of mind: An essay on faculty psychology.

[CR12] Forester G, Kroneisen M, Erdfelder E, Kamp S-M (2019). On the role of retrieval processes in the survival processing effect: Evidence from ROC and ERP analyses. Neurobiology of Learning and Memory.

[CR13] Forester G, Kroneisen M, Erdfelder E, Kamp S-M (2020). Survival processing modulates the neurocognitive mechanisms of episodic encoding. Cognitive, Affective, & Behavioral Neuroscience: CABN.

[CR14] Forester G, Kroneisen M, Erdfelder E, Kamp SM (2020). Adaptive memory: Independent effects of survival processing and reward motivation on memory. Frontiers in Human Neuroscience.

[CR15] Janczyk M (2017). A common capacity limitation for response and item selection in working memory. Journal of Experimental Psychology: Learning, Memory, and Cognition.

[CR16] Janczyk M, Kunde W (2020). Dual tasking from a goal perspective. Psychological Review.

[CR17] Janczyk M, Pfister R, Wallmeier G, Kunde W (2014). Exceptions from the PRP effect? A comparison of prepared and unconditioned reflexes. Journal of Experimental Psychology: Learning, Memory, and Cognition.

[CR18] Janczyk M, Renas S, Durst M (2018). Identifying the locus of compatibility-based backward crosstalk: Evidence from an extended PRP paradigm. Journal of Experimental Psychology: Human Perception and Performance.

[CR19] Janczyk M, Humphreys GW, Sui J (2019). The central locus of self-prioritization. The Quarterly Journal of Experimental Psychology.

[CR20] Jolicoeur P, Dell’Acqua R (1998). The demonstration of short-term consolidation. Cognitive Psychology.

[CR21] Kang SHK, McDermott KB, Cohen SM (2008). The mnemonic advantage of processing fitness-relevant information. Memory & Cognition.

[CR22] Kazanas SA, Altarriba J (2015). The survival advantage: Underlying mechanisms and extant limitations. Evolutionary Psychology.

[CR23] Kostic B, McFarlan CC, Cleary AM (2012). Extensions of the survival advantage in memory: Examining the role of ancestral context and implied social isolation. Journal of Experimental Psychology: Learning, Memory, and Cognition.

[CR24] Kroneisen M, Erdfelder E (2011). On the plasticity of the survival processing effect. Journal of Experimental Psychology: Learning, Memory, and Cognition.

[CR25] Kroneisen M, Erdfelder E, Buchner A (2013). The proximate memory mechanism underlying the survival-processing effect: Richness of encoding or interactive imagery?. Memory.

[CR26] Kroneisen M, Rummel J, Erdfelder E (2014). Working memory load eliminates the survival processing effect. Memory.

[CR27] Kroneisen M, Rummel J, Erdfelder E (2016). What kind of processing is survival processing? Effects of different types of dual-task load on the survival processing effect. Memory & Cognition.

[CR28] Kroneisen M, Kriechbaumer M, Kamp S-M, Erdfelder E (2021). How can I use it? The role of functional fixedness in the survival processing paradigm. Psychonomic Bulletin & Review.

[CR29] Kroneisen M, Kriechbaumer M, Kamp S-M, Erdfelder E (2022). Realistic context doesn’t amplify the survival processing effect: Lessons learned from Covid-19 scenarios. Acta Psychologica.

[CR30] Kunde W, Pfister R, Janczyk M (2012). The locus of tool transformation costs. Journal of Experimental Psychology: Human Perception and Performance.

[CR31] Miller J, Reynolds A (2003). The locus of redundant-targets and nontargets effects: Evidence from the psychological refractory period paradigm. Journal of Experimental Psychology: Human Perception and Performance.

[CR32] Nairne JS, Healy AF (2005). The functionalist agenda in memory research. Experimental cognitive psychology and its applications: Festschrift in honor of Lyle Bourne, Walter Kintsch, and Thomas Landauer.

[CR33] Nairne JS, Pandeirada JNS (2010). Adaptive memory: Ancestral priorities and the mnemonic value of survival processing. Cognitive Psychology.

[CR34] Nairne JS, Pandeirada JNS (2016). Adaptive memory: The evolutionary significance of survival processing. Perspectives on Psychological Science.

[CR35] Nairne JS, Thompson SR, Pandeirada JNS (2007). Adaptive memory: Survival processing enhances retention. Journal of Experimental Psychology: Learning, Memory, and Cognition.

[CR36] Nairne JS, Pandeirada JNS, Thompson SR (2008). Adaptive memory: The comparative value of survival processing. Psychological Science.

[CR37] Nairne JS, Coverdale ME, Pandeirada JNS (2019). Adaptive memory: The mnemonic power of survival-based generation. Journal of Experimental Psychology: Learning, Memory, and Cognition.

[CR38] Nouchi R (2012). The effect of aging on the memory enhancement of the survival judgment task. Japanese Psychological Research.

[CR39] Nouchi R (2013). Can the memory enhancement of the survival judgment task be explained by the elaboration hypothesis? Evidence from a memory load paradigm. Japanese Psychological Research.

[CR40] Nouchi R, Kawashima R (2012). Effect of the survival judgment task on memory performance in subclinically depressed people. Frontiers in Psychology.

[CR41] Otgaar H, Smeets T, Merckelbach H, Jelicic M, Verschuere B, Galliot A-M, van Riel L (2011). Adaptive memory: Stereotype activation is not enough. Memory & Cognition.

[CR42] Otgaar H, Jelicec M, Smeets T (2015). Identifying the proximate roots of the survival processing advantage. Journal of Psychology.

[CR43] Paelecke M, Kunde W (2007). Action-effect codes in and before the central bottleneck: Evidence from the PRP paradigm. Journal of Experimental Psychology: Human Perception and Performance.

[CR44] Pashler H (1994). Dual-task interference in simple tasks: Data and theory. Psychological Bulletin.

[CR45] Pietraszewski D, Wertz AE (2022). Why evolutionary psychology should abandon modularity. Perspectives on Psychological Science.

[CR46] Röer JP, Bell R, Buchner A (2013). Is the survival processing memory advantage due to richness of encoding?. Journal of Experimental Psychology: Learning, Memory, and Cognition.

[CR47] Rummel J, Steindorf L, Marevic I, Danner D (2019). A validation study of the German complex-span tasks and some general considerations on task translation procedures in cognitive psychology. European Journal of Psychological Assessment.

[CR48] Scofield JE, Buchanan EM, Kostic B (2018). A meta-analysis of the survival-processing advantage in memory. Psychonomic Bulletin & Review.

[CR49] Stillman CM, Coane JH, Profaci CP, Howard JH, Howard DV (2014). The effects of healthy aging on the mnemonic benefit of survival processing. Memory & Cognition.

[CR50] Telford CW (1931). The refractory phase of voluntary and associative responses. Journal of Experimental Psychology.

[CR51] Welford AT (1952). The “psychological refractory period” and the timing of high-speed performance: A review and a theory. British Journal of Psychology.

[CR52] Wickelgren WA (1977). Speed–accuracy tradeoff and information processing dynamics. Acta Psychologica.

[CR53] Wirth R, Pfister R, Janczyk M, Kunde W (2015). Through the portal: Effect anticipation in the central bottleneck. Acta Psychologica.

[CR54] Yang L, Lau KPL, Truong L (2014). The survival effect in memory: Does it hold into old age and non-ancestral scenarios?. PLOS ONE.

