# Supplementary material for: Survival processing occupies the central bottleneck of cognitive processing: A psychological refractory period analysis
Source: Psychon Bull Rev. 2023 Aug 11;31(1):274–82. doi: 10.3758/s13423-023-02340-z (PMC10867088; doi:10.3758/s13423-023-02340-z)
Supplement: Supplementary file 1 — Supplementary file1 (DOCX 42 KB) [file 13423_2023_2340_MOESM1_ESM.docx]

RUNNING HEAD:

SURVIVAL PROCESSING OCCUPIES THE CENTRAL BOTTLENECK

**ONLINE SUPPLEMENTAL MATERIAL**

Survival Processing Occupies the Central Bottleneck of Cognitive Processing:

A Psychological Refractory Period Analysis

Meike Kroneisen^1^, Edgar Erdfelder^2^, Rika Maria Groß^2^, & Markus Janczyk^3^

^1^Rheinland-Pfälzische Technische Universität Kaiserslautern Landau, Department of Psychology

^2^University of Mannheim, Department of Psychology

^3^University of Bremen, Department of Psychology

.

Correspondence concerning this article should be addressed to Meike Kroneisen, Department of Psychology, University of Koblenz-Landau, Fortstraße 7, D-76829 Landau, Germany. E-mail: [kroneisen@uni-landau.de](mailto:kroneisen@uni-landau.de)

The research reported in this article was supported by grants KR 4545/1-1 and ER224/3-1 of the Deutsche Forschungsgemeinschaft (DFG) to Meike Kroneisen and Edgar Erdfelder, respectively.

**Rating Response Times as a Function of Scenario and Relevance Ratings**

To assess possible moderator effects of rating outcomes on relevance rating RT1s for different scenarios, we ran an additional mixed ANOVA with rating RT1s as the dependent variable. Unfortunately, treating *Rating* as a 5-levels within-subject factor would result in losing more than 30 participants (i.e., almost one third of the analysis sample) due to an insufficient number of remaining RTs for at least one *Scenario*-*Rating* combination. We therefore decided to combine ratings within the “low” (1-3) versus “high” (4-5) rating levels, thus creating a within-subject factor *Rating Group* with two levels only. In this case, only one participant (from the survival scenario group) was excluded with less than six RTs in one cell.

**Results**

Figure OSM1 illustrates the sample means and standard errors of RT1 for the relevant conditions. Relevance rating RT1s were analyzed with a 2 × 2 mixed ANOVA using *Rating Group* (low vs. high) as a within-subject factor and *Scenario* (moving vs. survival) as a between-subject factor. For the moving scenario, observed mean RT1s were 1953 ms and 1974 ms for low and high ratings, respectively. For the survival scenario the corresponding mean RTs were 2132 and 2008 ms. Overall, the data yield significant main effects of scenario *F*(1,120) = 4.70, *p* = .032, η_p_² = .04, and of rating, *F*(1,120) = 7.62, *p* = .007, η_p_² = .06. The interaction was significant as well, *F*(1,120) = 15.56, *p* < .001, η_p_² = .11. While the simple main effect of rating was not significant for the moving scenario, *F*(1,61) = 0.57, *p* = .454, η_p_² = .01, it was significant for the survival scenario, *F*(1,59) = 25.90, *p* < .001, η_p_² = .31, with clearly longer mean RTs for low compared with high ratings.


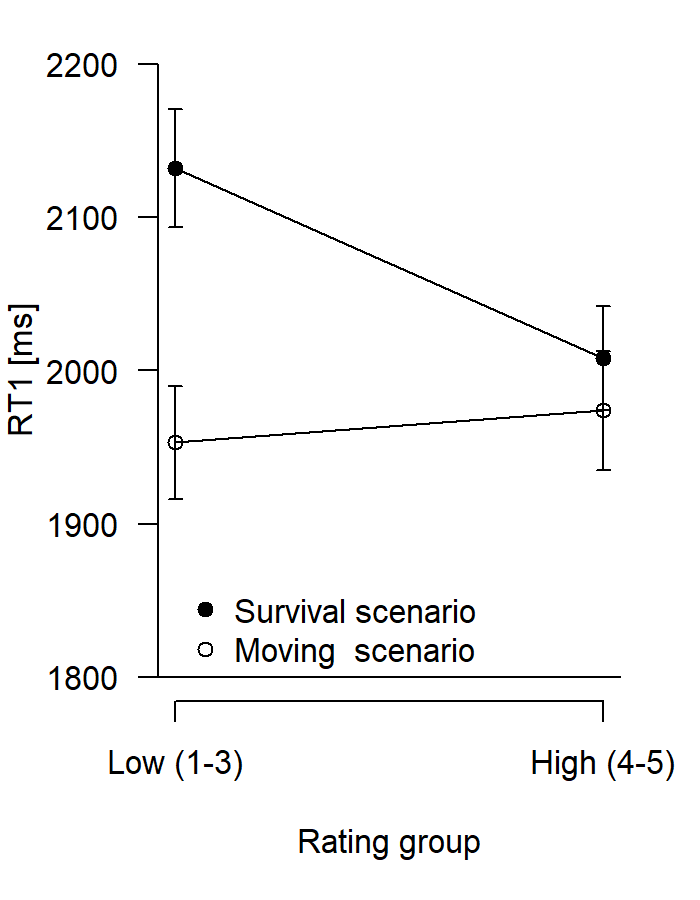


***Figure OSM1.*** Mean RT1s in milliseconds (ms) for relevance ratings (Task 1) as a function of scenario and rating group. Error bars indicate standard errors of the mean.

**Discussion**

Relevance rating RT1 differences between the survival and the moving scenario are substantial for low to medium relevance ratings and tend to diminish for high relevance ratings. In general, while relevance ratings RT1 for the moving scenario are barely affected by the rating group, they are considerably longer for low to medium survival relevance ratings. This is consistent with the idea that participants engage more strongly and persistently in identifying possible object functions when working under survival scenario instructions, especially when it is difficult to come up with reasonable ideas about object uses.
